# Time course and progression of wild type α-Synuclein accumulation in a transgenic mouse model

**DOI:** 10.1186/1471-2202-14-6

**Published:** 2013-01-09

**Authors:** David Amschl, Jörg Neddens, Daniel Havas, Stefanie Flunkert, Roland Rabl, Heinrich Römer, Edward Rockenstein, Eliezer Masliah, Manfred Windisch, Birgit Hutter-Paier

**Affiliations:** 1 QPS Austria GmbH, Parkring 12, Grambach, 8074, Austria; 2 Karl Franzens University, Institute of Zoology, Graz, 8010, Austria; 3 Department of Pathology, University of California San Diego, La Jolla, CA, USA

**Keywords:** Behavior, Immunofluorescence, Motor deficit, Mouse model, Parkinson’s disease, Phosphorylation, Synucleinopathy, α-Synuclein, Transgene

## Abstract

**Background:**

Progressive accumulation of α-synuclein (α-Syn) protein in different brain regions is a hallmark of synucleinopathic diseases, such as Parkinson’s disease, dementia with Lewy bodies and multiple system atrophy. α-Syn transgenic mouse models have been developed to investigate the effects of α-Syn accumulation on behavioral deficits and neuropathology. However, the onset and progression of pathology in α-Syn transgenic mice have not been fully characterized. For this purpose we investigated the time course of behavioral deficits and neuropathology in PDGF-β human wild type α-Syn transgenic mice (D-Line) between 3 and 12 months of age.

**Results:**

These mice showed progressive impairment of motor coordination of the limbs that resulted in significant differences compared to non-transgenic littermates at 9 and 12 months of age. Biochemical and immunohistological analyses revealed constantly increasing levels of human α-Syn in different brain areas. Human α-Syn was expressed particularly in somata and neurites of a subset of neocortical and limbic system neurons. Most of these neurons showed immunoreactivity for phosphorylated human α-Syn confined to nuclei and perinuclear cytoplasm. Analyses of the phenotype of α-Syn expressing cells revealed strong expression in dopaminergic olfactory bulb neurons, subsets of GABAergic interneurons and glutamatergic principal cells throughout the telencephalon. We also found human α-Syn expression in immature neurons of both the ventricular zone and the rostral migratory stream, but not in the dentate gyrus.

**Conclusion:**

The present study demonstrates that the PDGF-β α-Syn transgenic mouse model presents with early and progressive accumulation of human α-Syn that is accompanied by motor deficits. This information is essential for the design of therapeutical studies of synucleinopathies.

## Background

Synucleinopathic diseases, like Parkinson’s disease (PD), dementia with Lewy bodies (DLB) and multiple system atrophy (MSA), are all characterized by a pathologic aggregation of α-Synuclein (α-Syn) protein in distinct brain regions (reviewed by [1]). Increased expression of α-Syn can be caused by a dominant heritable form of PD due to duplication or triplication of the α-Syn gene [2-4]. In order to model such synucleinopathies *in vivo* different mouse models were developed that overexpress human wild type α-Syn (hα-Syn) [5-12]. When expression is driven by the murine Thy1 promoter, transgenic mice accumulate wild type hα-Syn in cortical and subcortical regions including the nigrostriatal system [6,13] whereas under the human PDGF-β promoter hα-Syn accumulates preferentially in the neocortex and limbic system (D-Line) [5].

Abnormal accumulation of hα-Syn in D-Line transgenic mice is accompanied by alterations in mGluR5 and autophagy similar to what has been observed in patients with dementia with Lewy bodies [14,15]. Moreover the behavioral and neurodegenerative pathology in D-Line mice can be reversed with mGluR5 antagonists [15] or by promoting the clearance of α-Syn with rapamycin [14], Beclin 1 [16,17] and neurosin [18]. Given the behavioral phenotype and the predominant accumulation of α-Syn in the neocortex and limbic system, these studies suggest that the PDGF-β hα-Syn transgenic mouse model reproduces some aspects of synucleinopathies such as DLB. Recent studies have tested compounds developed to ameliorate PD-like pathology in the D-Line mouse model and were able to show a reduction in the accumulation of α-Syn, total and oxidized α-Syn levels and behavioral deficits [19-22].

Taken together, these results indicate that this transgenic model might be useful for studies of α-Syn target validation. However, the progression of the behavioral deficits and pathology in D-Line mice has not been fully characterized. For this purpose we investigated the time course of behavioral deficits and neuropathology in D-Line mice. The occurrence of motor deficits was investigated using a challenging beam walk paradigm. We also quantified hα-Syn expression in the hippocampus and striatum using a biochemical approach. Moreover, by quantitative immunofluorescence at different ages we compared expression levels of both murine α-Syn and hα-Syn in the cortex, hippocampus, striatum and substantia nigra relative to non-transgenic littermates. Furthermore, we identified several cell populations that express transgenic hα-Syn in different areas of the adult brain.

## Results

### Progressive motor deficits of D-Line mice

**Figure 1:**
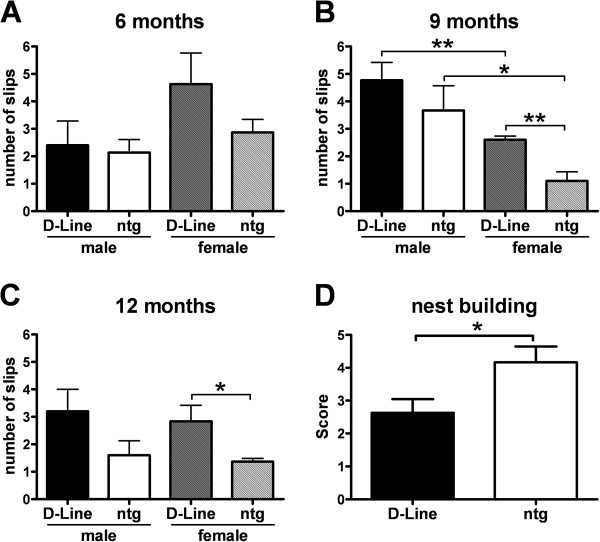
**D-Line mice present impaired motor coordination.** The number of slips by male and female D-Line mice compared to non-transgenic littermates were measured with the challenging beam walk test in 6 **(A)**, 9 **(B)** and 12 **(C)** months old individuals. Graphs show mean ± SEM; t-test. When analyzed by one way or two way ANOVA followed by Bonferroni’s *post hoc* test*,* factor transgene, no significant changes were observed. **(A-C)** D-Line male: n = 6; D-Line female: n = 6; wt male: n = 6; wt female: n = 6. **(D)** Pooled data of either sex on nest building behavior of D-Line mice compared to non-transgenic littermates in 9 months old individuals. Graph represents the mean nest building score (scoring from 1–5). D-Line (n = 8); ntg (n = 6). Graph shows mean ± SEM; Mann–Whitney U-test. * p < 0.05; ** p < 0.01.

Motor coordination, in particular of the hind limbs, and balance of all mice was evaluated with the challenging beam walk test at 6, 9 and 12 months of age to evaluate progression of motor deficits in adult, mature and aging mice. The time to cross the beam, the number of steps and slips, and the ratio of slips per step was recorded. No significant changes in the number of slips were observed in 6 months old D-Line and control animals (Figure 1A). 9 months old transgenic and non-transgenic male mice made significantly more slips compared to the corresponding female mice (Figure 1B). Transgenic female mice of the same age performed significantly worse compared to the corresponding female non-transgenic controls as analyzed by t-test. The same trend was observed in male mice, but the results were not statistically significant (Figure 1B). The observed deficits faded at the age of 12 months and only the significant difference between female D-Line and non-transgenic mice remained (Figure 1C). The significant differences shown in Figure 1A-C were detected by analyzing data with t-test. Analyses of the presented data for the factor gender by two way ANOVA followed by Bonferroni’s *post hoc* test revealed a significant influence of the gender for transgenic as well as non transgenic mice (p < 0.05, data not shown). Analyses of data by two way ANOVA followed by Bonferroni’s *post hoc* test for the factor transgene and by one way ANOVA did not reveal any significances. Analysis of the number of slips for progressive changes over age separated by sex showed only a significant improvement of non-transgenic female mice over age (two way ANOVA followed by Bonferroni’s *post hoc* test, factor time; data not shown). The time and number of steps required to traverse the beam and the ratio of slips per step did not differ significantly between D-Line and non-transgenic littermates (two way ANOVA, one-way ANOVA and t-test). In order to verify that the observed motor differences between the D-Line mice and non-transgenic littermates were not caused by modifications of spontaneous activity or deficits in spatial learning, the cylinder test and the two-choice swim test were performed, and no significant effects were detected (data not shown). General health of each mouse was determined using the Irwin test—including the wire hanging test and vertical pole test, assessing physical characteristics and abnormalities, sensorimotor reflexes and motor abilities—but no significant differences were observed. Overall, all animals appeared healthy over the course of the study.

The nest building test was used to analyze D-Line mice compared to non-transgenic littermates at 9 months of age. D-Line mice showed a significantly reduced nest building behavior (Figure 1D). While non-transgenic mice built an almost perfect nest with the provided material, the D-Line mice shredded only about 50% of the nesting material and an easily identifiable nest site was missing. Since no significant differences between male and female D-Line mice were observed (data not shown), data of both sexes were pooled for Figure 1D. Taken together, our behavioral analysis argues for an impairment of motor coordination in transgenic mice during execution of challenging tasks, whereas overall motor activity is largely unaffected.

### Quantification of α-synuclein protein levels over age

**Figure 2:**
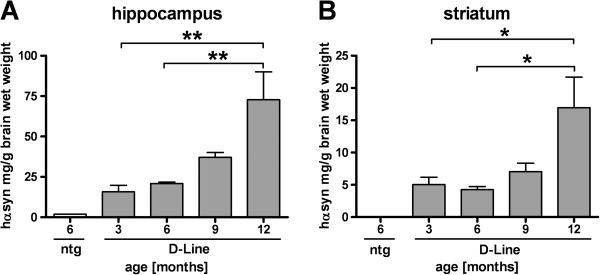
**Increasing transgenic α-Synuclein protein levels in the brain of 3–12 months old D-Line mice.** ELISA was used to analyze levels of hα-Syn in brain homogenates. Panels show hα-Syn expression levels of 3, 6, 9 and 12 months old D-Line mice in the hippocampus **(A)** and striatum **(B)** and 6 months old non-transgenic littermates. D-Line mice: 3, 9 and 12 months old (n = 6), 6 months (n = 5), ntg (n = 1). Values of one animal were excluded by Grubb’s Outlier test. Striatum ntg value = 0. Data of the non-transgenic mouse were not included in statistical analyses. Graphs show mean ± SEM; one-way ANOVA followed by Bonferroni’s *post hoc* test. * p < 0.05; ** p < 0.01.

**Figure 3:**
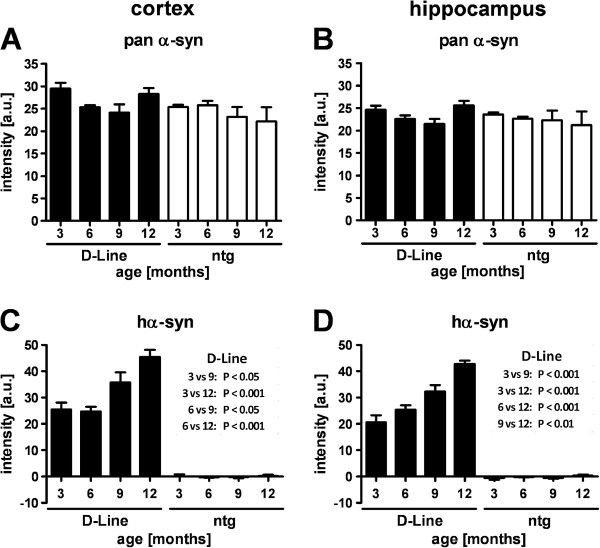
**Quantitative analyses of α-Synuclein levels in cerebral cortex of 3–12 months old mice.** Immunofluorescence intensity of pan α-Syn labeling **(A, B)** and transgenic hα-Syn labeling **(C, D)** in the cortex *(left panels)* and hippocampus *(right panels)*. The expression levels of pan α-Syn are constant throughout life and do not differ between genotypes, whereas hα-Syn expression in D-Line mice doubles between 3 and 12 months of age. D-Line: 3 months (n = 8), 6 months (n = 9), 9 months (n = 6), 12 months (n = 8); ntg mice: 3 months (n = 4), 6 months (n = 4), 9 months (n = 5), 12 months (n = 3). Graphs show mean ± SEM; one-way ANOVA followed by Bonferroni’s *post hoc* test.

**Figure 4:**
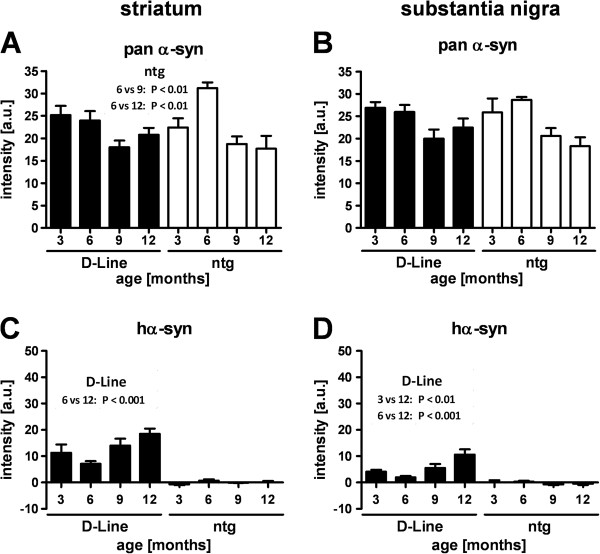
**Quantitative analyses of α-Synuclein levels in nigrostriatal areas of 3–12 months old mice.** Immunofluorescence intensity of pan α-Syn labeling **(A, B)** and transgenic hα-Syn labeling **(C, D)** in the dorsal striatum *(left panels)* and substantia nigra pars compacta *(right panels)*. The expression levels of pan α-Syn are relatively constant throughout life and do not differ between genotypes, whereas hα-Syn expression in D-Line mice increases between 3 and 12 months of age. D-Line: 3 months (n = 8), 6 months (n = 9), 9 months (n = 6), 12 months (n = 8); ntg mice: 3 months (n = 4), 6 months (n = 4), 9 months (n = 5), 12 months (n = 3). Graphs show mean ± SEM; one-way ANOVA followed by Bonferroni’s *post hoc* test.

Analyses of hα-Syn protein levels by ELISA showed a significant increase in the hippocampus at 12 months of age compared to 3 and 6 months old D-Line mice (Figure 2A). The increase was less pronounced in the striatum but was significant at 12 months compared to 3 and 6 months (Figure 2B). In order to investigate whether the progressive accumulation of hα-Syn in brain homogenates using ELISA could also be detected *in situ* we next analyzed expression levels of human and murine protein isoforms in the cortex, hippocampus, striatum and substantia nigra by quantification of immunofluorescent labeling using isoform-specific antibodies. In all analyzed brain areas the pan (murine and human) α-Syn levels of D-Line mice did not differ from non-transgenic littermates, and in both genotypes the total protein levels did not change significantly over age in cerebral cortical (Figure 3A-B) and subcortical (Figure 4A-B) areas. However, analyses restricted to transgenic hα-Syn, the same isoform that was analyzed biochemically by ELISA, showed significantly higher immunofluorescence signal intensity (p < 0.001) in D-line mice compared to ntg mice already at 3 months of age in cortical areas (Figure 3C-D). Expression levels of hα-Syn in the striatum and the substantia nigra were also above baseline in D-line mice (Figure 4C-D) but were generally lower than in cortical areas. Despite these regional differences in total levels, an age-dependent significant increase of hα-Syn immunofluorescence could be observed in all four brain areas of D-Line mice (Figures 3C-D, 4C-D), consistent with the data obtained by ELISA. This result, together with the absence of any age-related change in pan α-Syn, raises the possibility that adaptive mechanisms may downregulate murine α-Syn expression to ensure homeostatically appropriate protein levels throughout life. An alternative explanation for the finding of constant pan α-Syn levels is that the pan-specific monoclonal antibody may have lower affinity for the human isoform compared to murine α-Syn. This could potentially be assessed using an antibody that specifically detects only murine α-Syn, but to our knowledge such a reagent is not available.

### Distribution pattern of human, murine and phosphorylated α-synuclein protein

**Figure 5:**
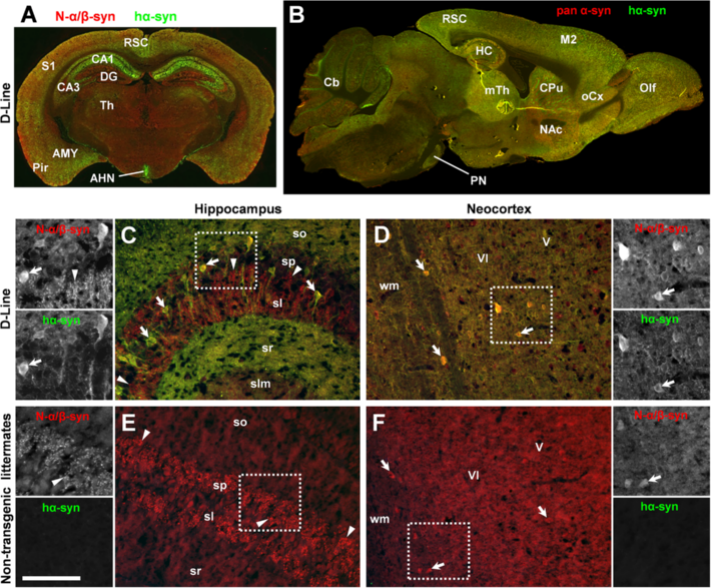
**Region-specific expression patterns of human α-Synuclein versus murine Synuclein in neocortical and hippocampal areas.** Coronal **(A)** and sagittal **(B)** overview images demonstrate that transgenic expression of hα-Syn *(green channel)* is most prominent in the cerebral cortex (RSC, S1, Pir, CA1-3, M2, oCx), subcortical telencephalic structures (AMY, CPu, NAc, Olf), medial thalamic nuclei (mTh), the arcuate hypothalamic nucleus (AHN), and the pontine nucleus (PN) in transgenic mice. Please note that expression in the hippocampal formation (HC) is restricted to CA1-3 but is largely absent from dentate gyrus (DG) **(A)**. In contrast, using antibodies directed against either **(A)** a species-independent N-terminal epitope of N-α/β-Syn or **(B)** a species-independent epitope of pan α-Syn shows expression throughout most areas of the brain including subcortical structures *(red channel)*. Higher magnification reveals accumulation of transgenic hα-Syn in a subset of cells (arrows) in the stratum pyramidale (sp) of hippocampal CA3 **(C)** and in cortical layers V-VI **(D)**. Neuropil in strata oriens (so) and radiatum (sr) shows high hα-Syn immunoreactivity, which in contrast is mostly absent arrowheads from strata lucidum (sl) and lacunosum moleculare (slm) **(C)**. In non-transgenic mice, hippocampal expression of murine α/β-Syn **(E)** is restricted to mossy fiber terminals in stratum lucidum (arrowheads) but is absent from neuronal somata, whereas in the neocortex **(F)** some cells close to white matter (wm) are α/β-Syn positive arrows. Please note the specificity of the antibody directed against the human isoform of α-Syn, as evident from absence of immunoreactivity on non-transgenic tissue *(green channel* in **E, F***)*. Scale bar: 2 mm **(A, B)**, 150 μm **(C-F)**. Abbreviations: amygdala (AMY), cerebellum (Cb), cornu ammonis areas 1–3 (CA1-3), caudate/putamen (CPu), motor cortex (M2), nucleus accumbens (NAc), orbital frontal cortex (oCx), olfactory bulb (Olf), piriform cortex (Pir), retrosplenial cortex (RSC), somatosensory cortex (S1), thalamus (Th).

Next we investigated the distribution patterns of hα-Syn and pan α-Syn isoforms by double-immunofluorescent labeling throughout the brain (Figure 5A-B). High levels of hα-Syn were detected in the pontine nucleus and in the cortex, including the cortical part of the amygdala and the hippocampus. High levels of hα-Syn accumulation were also detected in the striatum, ventral hypothalamus and in the olfactory bulb. Double-immunofluorescence for hα-Syn and a N-terminal epitope of both murine and human α/β-synuclein (N-α/β-Syn) at higher magnification revealed that transgenic hα-Syn obviously accumulates in somata and proximal dendrites. This is also evident in layers with few neuronal somata but high density of neuropil (Figure 5C-D), suggesting subcellular targeting of overexpressed hα-Syn to both dendrites and axons. In contrast, N-α/β-Syn is preferentially expressed in synaptic terminals, notably in the stratum lucidum of hippocampal CA3 (Figure 5C,E) and less obvious in the neocortex (Figure 5D, F), consistent with previous reports on largely presynaptic expression in wild type mice [23,24].

**Figure 6:**
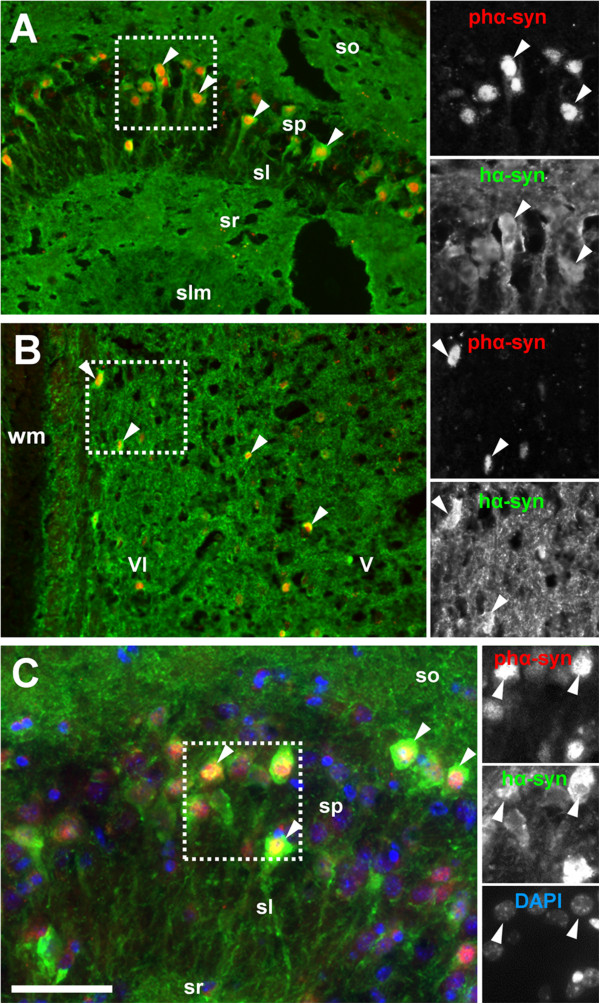
**Phosphorylated human α-Synuclein is targeted to nuclei but is undetectable in neurites.** Immunoreactivity for transgenic hα-Syn *(green channel)* is evident in a subset of pyramidal neurons **(A, C)** in the hippocampal area CA3 and **(B)** in the dorsolateral neocortex (arrowheads); in either area the transgenic isoform is targeted to both somata and neurites. Using an antibody against human protein phosphorylated at S129 (phα-Syn, *red channel)*, immunoreactivity is strong in most neurons that are positive for hα-Syn but is spatially restricted to neuronal nuclei, as is evident from co-localization with DAPI (arrowheads) **(C)**. Scale bar: 150 μm **(A, B)**, 100 μm **(C)**. Abbreviations: cornu ammonis areas 1–3 (CA1-3), stratum oriens (so), stratum pyramidale (sp), stratum lucidum (sl), stratum radiatum (sr), stratum lacunosum moleculare (slm), white matter (wm), cortical layer V and VI (V and VI).

Next, we investigated the expression pattern of phosphorylated protein (Figure 6) using an antibody specifically directed against an epitope including the phosphorylated-Serine 129 residue of the human α-Synuclein isoform (pS129-hα-Syn). Phosphorylation at residues S87 and S129 is of particular interest for synucleinopathy research because it has been argued that phosphorylation at these residues might modulate the formation of protein aggregations, e.g. inclusion bodies and fibrillary structures [25-27]. We found that in the hippocampus and neocortex, the subcellular expression of phα-Syn is largely restricted to neuronal nuclei (Figure 6), as evident from the overlay with the nuclear dye DAPI (Figure 6C). Qualitative analysis of pS129-hα-Syn suggests that the vast majority of neuronal somata expressing the hα-Syn isoform show easily detectable levels of pS129-hα-Syn. However, we found no evidence for expression in synaptic terminals or pathological intracellular accumulations in D-Line mice of up to 9 months. In contrast to the pS129S residue, phosphorylated S87 hα-Syn is undetectable in D-Line mice [27].

### Identification of α-synuclein-expressing neurons

**Figure 7:**
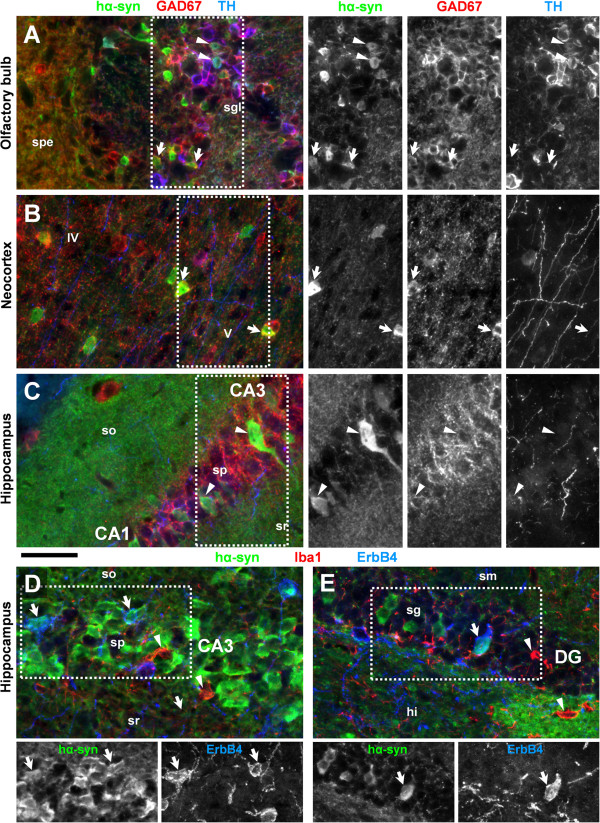
**Region-specific expression of human α-Synuclein in different subpopulations of neurons.** Triple immunofluorescent labeling reveals expression of transgenic hα-Syn *(green channel)* in different types of neurons. **(A)** hα-Syn immunoreactivity is evident in a subset (arrowheads) of dopaminergic neurons (TH, *blue channel*) in the glomerular layer (sgl), and occasionally (arrows) in GABAergic interneurons (GAD67, *red channel*) throughout the olfactory bulb. **(B)** A subset of neocortical hα-Syn-positive neurons (arrows) are immunoreactive for the interneuron marker GAD67 *(red channel)*; however, this did not occur in the hippocampus (arrowheads) probably due to known low expression levels of GAD67 in some types of interneurons **(C)**. Using ErbB4 immunoreactivity as an alternative marker for GABAergic neurons, we found hα-Syn expression in a subset of interneurons (arrows), but not in Iba1-immunoreactive microglia (arrowheads), in hippocampal area CA3 **(D)** and in the dentate gyrus **(E)**. Abbreviations: dentate gyrus (DG), glutamic acid decarboxylase 67 kD (GAD67), tyrosine hydroxylase (TH), stratum plexiforme externum (spe), stratum oriens (so), stratum pyramidale (sp), stratum radiatum (sr), stratum moleculare (sm), stratum granulosum (sg), hilus (hi) cortical layer V and VI (V and VI), cornu ammonis area 1/3 (CA1/3). Scale bar = 50 μm.

**Figure 8:**
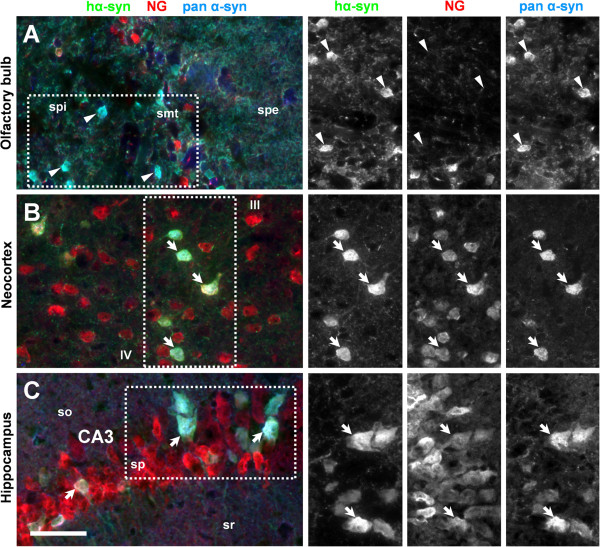
**Expression of human α-Synuclein in populations of principal neurons.** Triple fluorescent labeling reveals that immunoreactivity of both hα-Syn *(green channel)* and pan α-Syn *(blue channel)* is absent from neurogranin-positive mitral cells (NG, *red channel)* in the olfactory bulb (arrowheads in **A**). In contrast, a subset of neocortical **(B)** and hippocampal **(C)** pyramidal neurons expresses α-Syn; co-localization of all three color channels (arrows) is indicated by the occurrence of whitish pixels. Single channel images are rotated clockwise in panels **A** and **C**. Abbreviations: neurogranin (NG), stratum plexiforme externum/internum (spe/i), stratum mitrale (smt), stratum oriens (so), stratum pyramidale (sp), stratum radiatum (sr), cortical layers III and IV (III and IV), cornu ammonis area 3 (CA3). Scale bar = 50 μm.

**Figure 9:**
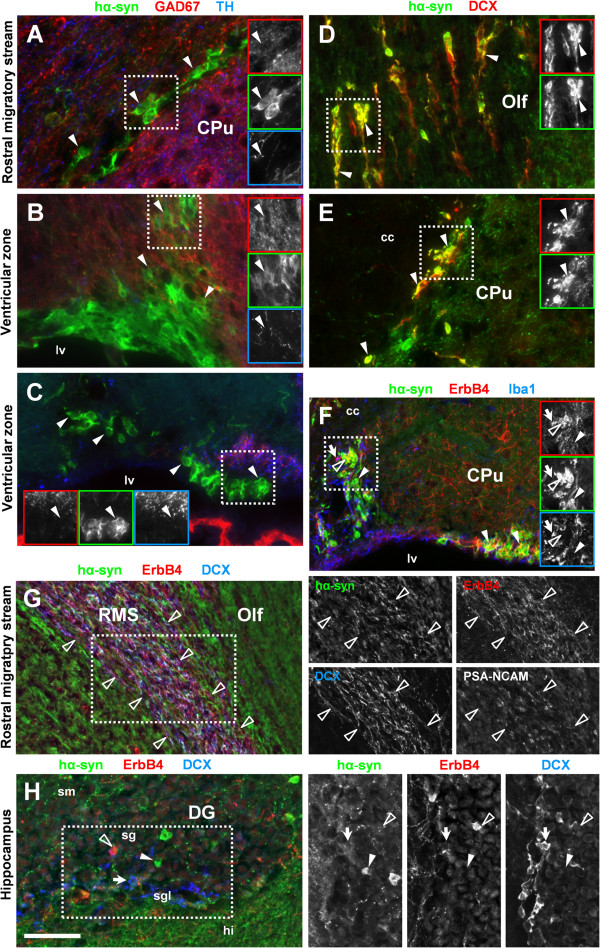
**Human α-Synuclein is strongly expressed in immature neurons in a region-specific pattern.** Immunofluorescent labeling of hα-Syn *(green channel)* in different regions of the brain shows strong expression of transgenic α-Syn (arrowheads) in immature neurons in the rostral migratory stream (RMS) **(A, D, G)** and in the ventricular zone (VZ) **(B, C, E, F)**. We found no coexpression with either GAD67 or TH **(A, B, C)**, whereas many hα-Syn-positive cells were immunoreactive for doublecortin (DCX) in the RMS **(D)** and the VZ **(E)**, indicating that hα-Syn is expressed in subpopulations of immature migrating neurons. **(F)** We also found co-expression of hα-Syn and ErbB4 in the VZ (open arrowhead in **F**) and the RMS **(G)**, whereas hα-Syn was not expressed in microglia immunoreactive for Iba1 (arrow in **F**). The VZ and the RMS were identified by immunoreactivity for DCX, ErbB4, or PSA-NCAM **(G)**. **(H)** In contrast to the VZ and RMS, we found no indication for expression of either hα-Syn (arrowhead) or ErbB4 (open arrowhead) in DCX-positive immature neurons (arrow) in the subgranular layer (sgl) of the hippocampal dentate gyrus (DG). Single channel images are rotated clockwise in panel H. Abbreviations: caudate/putamen (CPu), corpus callosum (cc), glutamic acid decarboxylase 67 kD (GAD67), hilus (hi), lateral ventricle (lv), olfactory bulb (Olf), stratum granulosum (sg), stratum moleculare (sm), tyrosine hydroxylase (TH), doublecortin (DCX). Scale bar = 50 μm **(A-E, H)**, 70 μm **(F, G)**.

In order to identify neuronal populations that accumulate hα-Syn, several multichannel immunofluorescence experiments were performed. Using antibodies against GAD67 and tyrosine hydroxylase we found that hα-Syn is expressed in subpopulations of GABAergic and dopaminergic neurons, respectively, of the olfactory bulb (Figure 7A), and in GABAergic interneurons of cortical areas (Figure 7B-C). Since some GABAergic somata in the hippocampal formation show little or even no GAD67 immunoreactivity [28] we alternatively identified interneurons by expression of the neuregulin receptor ErbB4 that is a selective marker for a subset of cortical GABAergic neurons [29-31]. We detected hα-Syn in a small number of ErbB4-positive interneurons in the cornu ammonis and dentate gyrus but not on microglia identified by Iba1 immunoreactivity (Figure 7D). With regard to glutamatergic principal neurons, hα-Syn was not detectable in mitral cells of the olfactory bulb (Figure 8A), but strong expression was evident in subsets of neocortical and hippocampal pyramidal cells (Figure 8B-C). We noted the occurrence of strong hα-Syn immunoreactivity in some regions of the adult brain featuring immature and migrating neurons (Figure 9A-G), such as the ventricular zone and the rostral migratory stream. Using multichannel immunofluorescence, we confirmed hα-Syn expression in immature migrating neurons that were identified by several established markers such as doublecortin, PSA-NCAM and ErbB4 ( [32-36]. In contrast, hα-Syn immunoreactivity was consistently absent from immature neurons in the hippocampal dentate gyrus (Figure 9H), suggesting that hα-Syn expression is not a unifying feature of migrating or maturing neurons in adult D-Line mice. Taken together, our data show that strong hα-Syn immunofluorescence occurs region-specifically in heterogeneous populations of neurons of the adult brain.

## Discussion

This study was designed to characterize the time course and progression of the pathology in D-Line mice, a model of α-Syn accumulation similar to DLB. We found progressive motor coordination deficits at 9 and 12 months of age complemented by alterations in the nest building behavior. These deficits were accompanied by a parallel age-dependent increase in the levels of hα-Syn in somata and neuritic processes of a subset of neocortical, limbic and nigrostriatal system cells. The mechanisms by which accumulation of hα-Syn in these circuitries might result in functional deficits are not completely clear. Recent findings on changes in expression levels of neurotrophic factors in hα-Syn transgenic mice [37] suggest that altered BDNF levels could be linked to onset and progression of Parkinson’s disease [38].

The progressive motor alterations in the challenging beam walk test detected in D-Line mice are consistent with previous studies using the rotarod and pole test [5,15] and support a dysfunction of the nigrostriatal system. Also supporting this possibility, previous studies have shown that D-Line mice of either sex generally perform worse compared to non-transgenic littermates; however, at the age of 9 months D-Line and non-transgenic male mice consistently slip more often during the challenging beam walk test than females. In transgenic mice the sex difference seems to increase up to the age of 9 months, suggesting that hα-Syn has an earlier impact on motor performance in male than in female mice. Interestingly, these results are reminiscent of sex differences in humans where female Parkinson patients show a delayed onset of the disease compared to males [39].

Nest building behavior is an intrinsic behavior in both female and male mice that requires proper fine motor skills. The nest is important for sustaining body temperature and provides shelter during birth and rearing of offspring. Different lesioning studies provide evidence that nest building behavior strongly depends on the proper function of the hippocampus, caudate putamen and ventral mesencephalic tegmentum [40-42]. Moreover, nest building was shown to be dopamine- and enkephalin-dependent [43]. All these brain systems are known to be highly relevant for PD [44,45], suggesting that the nest building test is an appropriate tool for assessing Parkinson-associated impairments. Therefore, our here presented data of nest building and motor deficits in the beam walk test suggest, that D-Line transgenic mice represent with the most common behavioral symptoms of PD.

Analyses of hα-Syn levels in the brain by ELISA showed an age dependent increase of transgenic protein levels in the hippocampus and striatum between 3 and 12 months. Using quantitative analysis of immunofluorescence against hα-Syn we could verify this result *in situ*. Interestingly, however, we detected neither genotype-dependent nor age-dependent increases in total (human and murine) α-Syn levels. Given that under the PDGF-β promoter the mRNA expression of α-Syn is stable throughout life, the progressive accumulation of hα-Syn protein in the brains of the transgenic mice indicates deficits in clearance. This is consistent with previous studies showing alterations in the autophagy pathway in the D-line mice [14,46], these alterations were reversed pharmacologically with rapamycin [14] or genetically with Beclin-1 [17] or Atg7 [14] and worsen with shAtg7 [14] or Bafilomycin-A1 [46]. In addition, the finding of constant total α-Syn levels despite the parallel accumulation of transgenic hα-Syn protein raises the possibility that the expression of endogenous murine α-Syn might be a tightly regulated mechanism. Overexpression of hα-Syn could therefore be a trigger for homeostatic downregulation of the murine isoform. Moreover, total α-Syn immunoreactivity even decreased slightly in the substantia nigra of non-transgenic animals suggesting that reduction of α-Syn levels in the nigrostriatal dopaminergic pathway of older mice is a normal event during aging in healthy rodents, consistent with reports by other groups [47,48].

We identified subsets of transgene expressing neurons as inhibitory GABAergic interneurons or excitatory glutamatergic pyramidal neurons in cortical areas. With respect to dopaminergic cells, we found co-expression of hα-Syn and tyrosine hydroxylase in many neurons of the olfactory bulb, in contrast to the substantia nigra where only low levels of hα-Syn were detectable. We also detected hα-Syn immunoreactivity in some GABAergic interneurons of the olfactory bulb but, in contrast to cortical areas, not in glutamatergic principal neurons (mitral cells). Overall, this pattern is consistent with the reported expression of PDGF-β in different types of neurons [49,50]. Our investigation of hα-Syn phosphorylation at residue S129 shows that immunoreactivity is largely restricted to the nuclei of neurons that express high levels of the transgenic protein. Of note, we found no indication of granular or fibrillary-like accumulations of pS129-hα-Syn, opening the question whether this nuclear immunoreactivity reflects regular physiological events or rather indicates progression towards neuropathology consistent with previous reports [51]. Finally, consistent with previous studies [52] we found that hα-Syn accumulates in areas featuring immature neurons, such as the subventricular zone and the rostral migratory stream. Accumulation of hα-Syn in these areas results in reduced proliferation and survival of neuroblasts [52], however, the functional relevance of these alterations is yet unknown. Interestingly, we found no indication of hα-Syn immunoreactivity in maturing neurons of the dentate gyrus, indicating that hα-Syn expression is not a common feature of all populations of immature or migrating neurons. Additional studies are required to investigate whether these regional differences have functional relevance that may affect survival rates or maturation of these cells.

## Conclusions

Our data suggest that hα-Syn accumulates in subsets of glutamatergic, dopaminergic, and GABAergic, neuronal populations in the neocortex, limbic system and nigrostriatal system similar to the distribution of hα-Syn in DLB. Immunoreactivity for human protein phosphorylated at residue S129 is restricted to neuronal somata, whereas much of the additional immunofluorescent signal from hα-Syn protein not phosphorylated at the S129 residue comes from areas with few neuronal somata but dense neuropil, thus mimicking the distribution of the endogenous murine α-Syn isoform. We also detected progressively increasing levels of hα-Syn protein during adulthood using different experimental approaches, and concomitant impairment of motor coordination. In contrast, the level of total α-Syn is constant between 3–12 months, possibly due to a compensatory downregulation of the endogenous α-Syn isoform. We conclude that different readouts, i.e. behavioral, biochemical and histological, can detect structural and functional pathology in the D-Line mouse model, and that this animal model is highly valuable for PD-related research and drug development.

## Methods

### Animals

For this study we used male and female mice over-expressing human wild type α-Syn under the regulatory control of the platelet-derived growth factor (PDGF-β) promoter with a C57BL/6xDBA background (D-Line, [5]). All experiments were performed with hemizygous D-Line mice and corresponding non-transgenic littermates. Animals were housed in individually ventilated cages on standardized rodent bedding (Rettenmayer®). The room temperature was kept at approximately 24°C and the relative humidity between 40-70%. Mice were housed under constant light-cycle (12 hours light/dark). Dried pelleted standard rodent chow (Altromin®) and normal tap water were available to the animals ad libitum. The health and well-being of each individual animal was monitored regularly. Animal studies conformed to the Austrian guidelines for the care and use of laboratory animals and were approved by the Styrian Government, Austria.

### Behavioral tests

For behavioral tests 6, 9 and 12 months old animals were used. 12 males (6 tg and 6 ntg) and 12 females (6 tg and 6 ntg) were analyzed per age group. The challenging beam walk test was carried out with all mice as previously described [53]. In short, mice were trained to traverse the length of a beam starting at the widest section and ending at the narrowest, most difficult section. The narrow end of the beam led directly into the animal’s home cage. Mice received two days of training before testing. In order to increase difficulty on the day of testing, a wire mesh of corresponding width was placed over the beam. On the testing day mice were required to run five trials. Mice were video-taped while traversing the grid-surfaced beam. Videotapes were viewed and rated in slow motion for slips, number of steps, slips per step and time to traverse. After normal distribution was verified by Kolmogorov-Smirnov test, group differences were calculated by one-way ANOVA, two-way ANOVA followed by Bonferroni’s *post hoc* test, factors transgene, gender and time, and unpaired t-test, using GraphPad Prism 4.03.

To test the individual nest building behavior, 9 months old mice were housed separately overnight in cages containing wood chip bedding and one 5 cm square piece of pressed cotton (nestlet). No other nesting material was provided. The nestlet was introduced on the day before the evaluation of the nest. The following morning the nest was assessed, according to a five-point scale [54]. If the nestlet was not noticeably touched it was scored with 1 point. A near perfect nest, in which at least 90% of the nestlet was used, was scored with 5 points. After normal distribution was unconfirmed by Kolmogorov-Smirnov test, group differences were calculated by Mann–Whitney U-test, using GraphPad Prism 4.03.

### Tissue preparation

Mice were deeply anesthetized by Isoflurane (BAXTER®, Austria) and the thorax was opened to excavate the heart. Animals were flush-perfused transcardially with 0.9% saline through the left ventricle. The hemispheres were divided at midline. The right hemisphere was immersion fixed in 4% paraformaldehyde in 0.1 M phosphate buffer, pH 7.4, for 1 h at room temperature (RT), cryoprotected in 30% sucrose, and snap-frozen in dry ice-cooled isopentane for further histological evaluations. The left hemisphere was dissected into hippocampus, striatum and rest brain, shock-frozen on dry ice, and stored at −80°C for biochemical hα-Syn determination.

### Biochemistry

We analyzed expression levels of hα-Syn in the hippocampus and striatum of D-Line mice and non-transgenic littermates at different ages: D-Line: 3, 9 and 12 months (n = 6), 6 months (n = 5); ntg: 6 months (n = 1). Total protein was extracted from brain samples by homogenization in 8 volumes guanidine buffer (5 M guanidine HCl, 50 mmol/L TrisHCl, pH 8.0). The homogenates were mixed for 4 h at RT. Samples were then diluted 500x (hippocampus), 100x (striatum) with cold reaction buffer (2.68 mmol/L KCl, 1.47 mmol/L KH_2_PO_4_, 136.89 mmol/L NaCl, 8.1 mmol/L Na_2_HPO_4_, 5% BSA, 0.03% Tween-20 and protease inhibitor cocktail) and centrifuged at 16.000 g for 20 min at 4°C. Supernatants were analyzed for total hα-Syn concentrations by ELISA (Hu α-Synuclein ELISA Kit #KHB0061, Invitrogen) following the manufacturer’s protocol. Group differences were statistically analyzed by excluding outliers using Grubb’s test and subsequent one-way ANOVA followed by Bonferroni’s *post hoc* test, using GraphPad Prism 4.03.

### Histology

We quantitatively investigated tissue obtained from mice at different ages (Figures 3– 4). D-Line / ntg: 3 months (n = 8 / 4); 6 months (n = 9 / 4); 9 months (n = 6 / 5); 12 months (n = 8 / 3). A natural α-Syn knockout line from Harlan (C57BL/6JOlaHsd) served as negative control to test for antibody specificity (Additional file 1).

Qualitative multichannel immunofluorescence experiments (Figures 5– 9) were performed at RT on sagittal cryosections (10 μm thick; systematic uniform random sampling) of adult mice (age 9 months, n = 3 per genotype, mixed sex) using the following protocol: Wash cryosections 2 × 5 min in 0.05 M tris-buffered saline (TBS, pH 7.6) with 0.25% Triton X-100, block 1 h with MOM blocking reagent (Vector, Burlingame, CA), wash 2 × 2 min, incubate 5 min in MOM diluent, incubate 40 h at 4°C in new MOM diluent with primary antibodies, wash 3 × 5 min, incubate secondary antibodies 1 h in MOM diluent wash 3 × 5 min, mount with Mowiol.

The expression of transgenic hα-Syn was detected with a rat monoclonal antibody (clone 15 G7) directed against residues 116–131 near the C-terminus of hα-Syn; this antibody does not bind to murine α-Syn. hα-Syn phosphorylated at Serine 129 (pS129-hα-Syn) was visualized with a rabbit monoclonal antibody (clone EP1536Y). We detected α/β-Synuclein expression using a goat polyclonal antibody raised against an N-terminal sequence of α-Syn of human origin (N-α/β-Syn); this antibody labels both murine and human isoforms of α/β-Syn. A pan-specific (pan α-Syn) mouse monoclonal antibody (clone 4D6) was used to detect both murine α-Syn and hα-Syn. Primary anti-α-Syn antibodies were tested for specificity on brain sections of α-Syn knockout mice as shown in a supplementary figure (Additional file 1). Detailed information on all primary antibodies used in this study is provided in Table 1.

**Table 1:** List of primary antibodies

**Species**	**Antigen**	**Clone**	**Source**	**Item #**	**Dilution**
rat	Human α-Synuclein	15 G7	Enzo Life Sciences, Plymouth Meeting, PA	804-258-L001	1:20
mouse	Human phospho α-Synuclein	EP1536Y	Abcam, Cambridge, UK	ab51253	1:2000
mouse	α-Synuclein	4D6	Abcam	ab1903	1:500
goat	α/β-Synuclein, N-terminal	poly	Santa Cruz Biotechnology, Santa Cruz, CA	sc-7012	1:300
goat	Doublecortin	poly	Santa Cruz Biotechnology	sc-8066	1:200
rabbit	Iba1/AIF1	poly	ProteinTech Group, Chicago, IL	10904-1-AP	1:500
rabbit	Tyrosine hydroxylase	poly	Novus Biologicals, Cambridge, UK	NB300-109	1:1000
rabbit	Neurogranin	poly	Millipore, Temecula, CA	AB5620	1:1000
mouse	GAD67	1 G10.2	Millipore	MAB5406	1:2000
mouse	PSA-NCAM	2-2B	Millipore	MAB5324	1:200
mouse	N-ErbB4	H4.77.16	LabVision, Fremont, CA	MS-270 (mAb-77)	3 μg/ml
rabbit	C-ErbB4	mAb-10	Buonanno Lab, NIH; Vullhorst et al., 2009	mAb-10	3 μg/ml

Secondary antibodies donkey anti-rat, donkey anti-mouse, donkey anti-rabbit, and donkey anti-goat were labeled with Cy2/DyLight488, Cy3, or Cy5/DyLight649 fluorophores (Jackson ImmunoResearch, West Grove, PA); all secondary antibodies were highly cross-adsorbed (ML quality) to prevent unspecific cross-reactivity. Specificity of secondary antibodies was assessed by omitting primary antibodies on parallel section as shown in an additional file (Additional file 1). Controls were routinely executed together with regular experiments.

Images for quantitative analysis were recorded with an Axio mRm camera mounted on AxioImager Z1 epifluorescence microscope at 10x magnification. Exposure time and additional settings were kept constant for all images used in quantification. Camera was set to the linear default. Regions of interest (ROI) were defined by individual delineation of the cortex, hippocampus and substantia nigra, and total α-Syn immunofluorescence signal was determined by integrating pixel intensity throughout the ROI using ImageProPlus Software (Version 6.2). Five sections per brain region were analyzed deriving from five different systematically chosen medio-lateral levels. After normal distribution was verified by Kolmogorov-Smirnov test, group differences in histological variables were calculated by one-way ANOVA followed by Bonferroni’s *post hoc* test, using GraphPad Prism 4.03. Epifluorescence z-stack images (8–10 z-levels, 0.3 μm apart, 20% overlay) presented in the figures were obtained at 40x magnification and were collapsed using the *Extended Focus* function in AxioVision (v4.8) software.

## Abbreviations

(hα-Syn): Human α-synuclein; (DLB): dementia with Lewy bodies; (DCX): doublecortin; (ErbB4): v-erb-a erythroblastic leukemia viral oncogene homolog 4; (GAD67): glutamic acid decarboxylase 67 kD; (Iba1/AIF1): ionized calcium binding adaptor molecule 1/allograft inflammatory factor 1; (MSA): multiple system atrophy; (ntg): non-transgenic; (PD): Parkinson’s disease; (phα-Syn): phospho-S129 human α-synuclein; (PSA-NCAM): polysialic acid-neuronal cell adhesion molecule; (ROI): region of interest; (TH): tyrosine hydroxylase.

## Competing interests

DA, DH, JN, SF, RR, MW and BHP are employees of QPS Austria GmbH.

## Authors’ contributions

Conceived and designed the experiments: JN, RR, HR, MW, BHP. Performed the experiments: DA, JN, RR. Analyzed the data: DA, DH, JN, RR. Contributed reagents/materials/analysis tools: ER, EM. Wrote the paper: SF, JN, EM, BHP. All authors read and approved the final manuscript.

## Supplementary Material

Additional file 1 **Negative controls for specificity of primary and secondary antibodies.** Specificity of (A) the monoclonal rat anti-human α-Syn antibody and (B) the pan-specific monoclonal mouse anti-human and anti-murine α-Syn antibody were tested on frontal sections through the neocortex (Cx), corpus callosum (cc) and caudate/putamen (CPu) of α-Syn knockout mice. We also tested specificity of secondary antibodies (C-E) on sections through the hippocampal formation of D-Line mice; the panels show residual fluorescence in the CA3 region after omitting primary antibodies. Only low level background fluorescent signal was detected in all control experiments. Abbreviations: stratum oriens (so), stratum pyramidale (sp), stratum radiatum (sr). Scale bar = 70 μm (A, B), 50 μm (C-E). Click here for file
