# Supplementary figures and images for: Time course and progression of wild type α-Synuclein accumulation in a transgenic mouse model
Source: BMC Neurosci. 2013 Jan 9;14:6. doi: 10.1186/1471-2202-14-6 (PMC3546911; doi:10.1186/1471-2202-14-6)

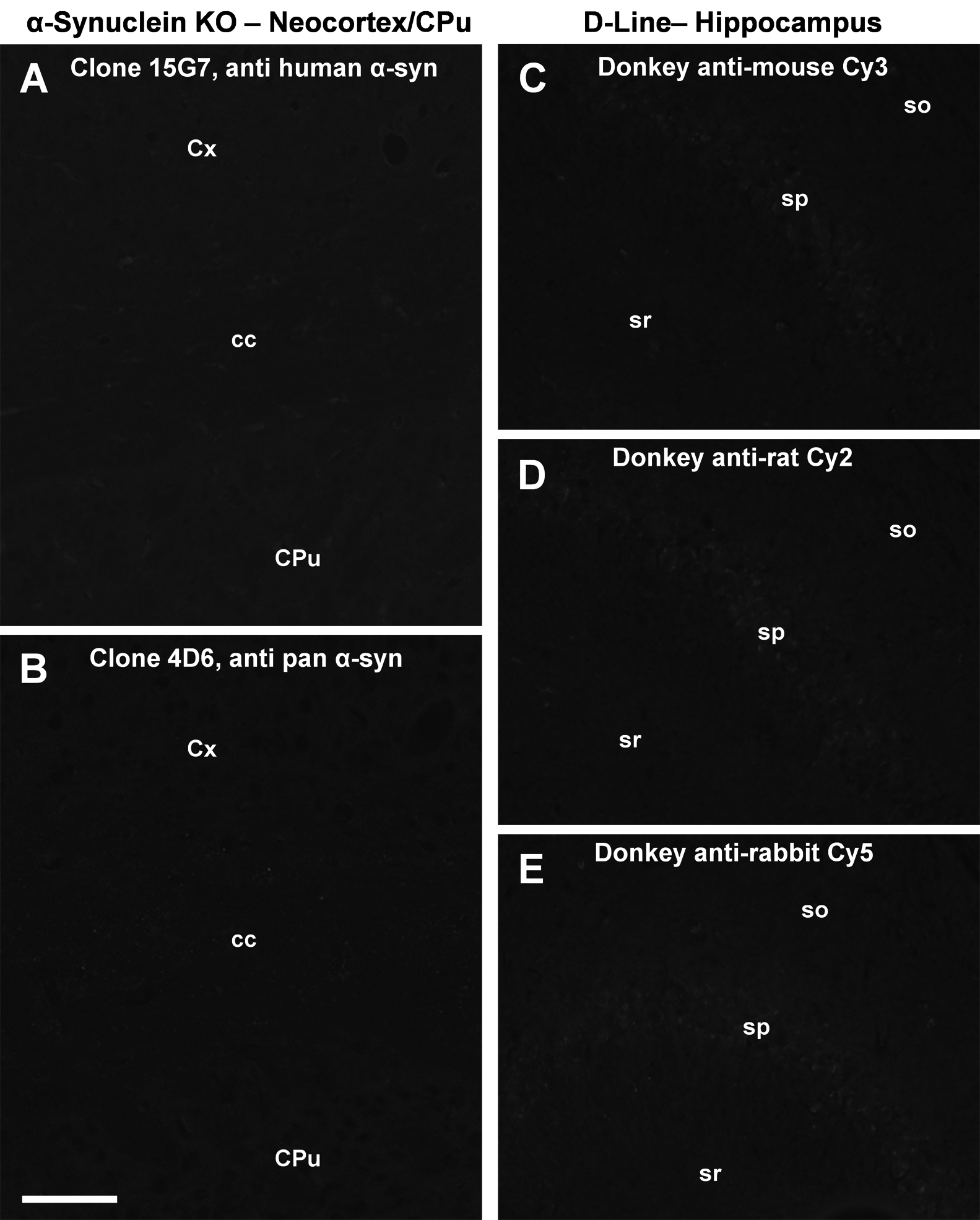

Supplement: Additional file 1 — Negative controls for specificity of primary and secondary antibodies. Specificity of (A) the monoclonal rat anti-human α-Syn antibody and (B) the pan-specific monoclonal mouse anti-human and anti-murine α-Syn antibody were tested on frontal sections through the neocortex (Cx), corpus callosum (cc) and caudate/putamen (CPu) of α-Syn knockout mice. We also tested specificity of secondary antibodies (C-E) on sections through the hippocampal formation of D-Line mice; the panels show residual fluorescence in the CA3 region after omitting primary antibodies. Only low level background fluorescent signal was detected in all control experiments. Abbreviations: stratum oriens (so), stratum pyramidale (sp), stratum radiatum (sr). Scale bar = 70 μm (A, B), 50 μm (C-E). [file 1471-2202-14-6-S1.png]
